# Innovating undergraduate pathology education through public engagement

**DOI:** 10.1007/s00428-018-2299-z

**Published:** 2018-02-27

**Authors:** Navin Mukundu Nagesh, Bogdan Chiva Giurca, Suzy Lishman

**Affiliations:** 1 Exeter Medical School, Heavitree Road, Exeter, EX1 2LU UK; 2 Royal College of Pathologists, 21 Prescot St, 4th Floor, London, E1 8BB UK

**Keywords:** Medical education, National Pathology Week, Public engagement

## Abstract

The trends in modern undergraduate medical education focus on a patient-centred approach through problem-based learning over the traditional modular curriculum. Integrating pathology into this style of learning has resulted in the dilution of core scientific principles which may have contributed to reduced understanding and interest in the subject. We aim to innovate pathology education by utilising National Pathology Week which is organised by the Royal College of Pathologists to develop the public engagement model which empowers students to learn pathology by teaching the public. Through this model, we hope to generate a greater interest in pathology at both undergraduate and postgraduate stages of education. We obtained funding from the Royal College of Pathologists to organise National Pathology Week at Exeter Medical School and the Royal Devon & Exeter Hospital. We involved 125 undergraduate student volunteers from health-related courses. We designed a curriculum aiming to educate both students and public on current topics such as cancer screening programmes, antibiotic resistance, diagnosis of inflammatory bowel disease and the role of pathologists. We hosted 15 pathologists, biomedical scientists and microbiologists to engage with students, share experiences and offer an insight into their careers. Through this project, we interacted with over 500 members of the public and 150 school students. The medical student volunteers developed a range of skills including competent use of microscopes to visualise pathology slides, effective communication with lay audiences to teach pathology and understanding of the clinical application of pathology. We believe the public engagement model of teaching undergraduate students has the potential to develop a greater interest in pathology whilst benefitting the wider community.

## Introduction

The word pathology is derived from the Greek words “*Pathos*” and “*Logos*” which can be translated into the study of suffering and illness. The origins of medical education have structured the learning of pathology through observation, rational interpretation of evidence and the development of hypotheses [1]. Rudolf Virchow, the father of pathology, said “*medical education does not exist to provide students with a way of making a living, but to ensure the health of the community*” [2]. This epitomises the core principles to which training should be structured for future doctors. Ensuring the health of the community can only be achieved with doctors that understand the origin, manifestations and progression of disease to adequately care for their patients. It can therefore be argued that a basic understanding of pathology is crucial for medical students for their development into tomorrow’s doctors.

The United Kingdom (UK) undergraduate medical curriculum has undergone a number of transformations within the last 15 years [3]. The development of new medical schools, expansion of cohort sizes and increasing expectations from graduates have resulted in fundamental changes in the structure of the medical curriculum. The traditional medical curriculum encompassing individual modular subject has been replaced by problem-based learning, system-based study and integrated learning methods [4, 5]. These changes ultimately emphasise clinical relevance and patient-centred focus over empirical study of basic biomedical modules such as pathology, biochemistry, pharmacology, etc. This paradigm shift can be attributed to the dilution of pathology teaching observed in modern medical schools. The formerly practical educational aids such as cadaveric dissection, microscopy and learning from preserved specimens are being replaced with the use of plastinated models and technology through medical imaging to develop an integrated pathology education [6]. Some medical schools have removed formal examinations in pathology and have substituted them with clinical case-based questions [7]. Numerous studies have shown that the “*testing effect*” through examinations is the most effective method of knowledge uptake and retention in education [8]. The lack of formal examinations in pathology may yield qualified doctors who are unaware of the basic principles of disease but are deemed competent in its management.

The net result of the changes of the undergraduate medical curriculum for pathology teaching may contribute to the development of misconceptions and ignorance regarding what a career in pathology entails. A qualitative analysis of the revised medical curriculum at Guy’s, King’s, St. Thomas School of Medicine revealed that 5th year students reported a worrying lack of understanding in pathology and therapeutics [9]. Incidences such as this could potentially deter junior doctors from pursuing a career in pathology due to uncertainty and lack of understanding. The competition ratios for applications for specialty training in pathology in the UK between 2012 and 2016 range from 1.3–2.4 [10]. This ratio consistently represents the lower end of the spectrum compared to other specialities. The cause of the relatively low number of applications cannot wholly be attributed to the changing medical curriculum. Trends towards quality of work life balance and flexibility may also attract applicants towards training programmes of shorter duration and fewer examinations [11].

The Royal College of Pathologists (RCPath) of the UK published figures from a workforce census for 2016 [12]. It reported a 5% reduction in the number of consultants aged over 55 within pathology specialities compared to the previous year. This can be attributed to early retirements and results in insufficient trainees to fill the vacant posts. Clinical Biochemistry for example was reported to have 162 consultants nationally, which was insufficient to continue providing a consultant-led service. More worryingly, there are currently only 51 trainees in Clinical Biochemistry, which is therefore insufficient to replace the retiring consultants in the future. The RCPath has developed a number of innovative schemes to increase awareness of pathology by targeting a wide range of audiences from children to medical trainees. National Pathology Week (NPW) represents an annual celebration of the invaluable contribution pathologists make to healthcare. The week comprises of public engagement activities delivered by healthcare professionals to educate and raise awareness of the science behind the cure.

The RCPath has successfully run NPW for a number of years targeting a wide range of audiences [13]. These events have historically been led by pathologists, trainees and scientists with undergraduate students only recently joining the extended team. We propose to innovate undergraduate pathology education by developing the public engagement model to empower students to learn pathology by teaching the public. This student-led approach provides a platform to run public engagement activities, whilst learning the basic principles of pathology, as well as the roles of pathologists within healthcare. We propose this novel model of pathology education to actively involve students in pathology-related activities to ultimately work towards ensuring the health of the community.

## Materials and methods

### Organisations and student volunteers

The Exeter Medical Leadership & Management Society organised the public engagement activities during NPW 2016 (November 7–11th). This society is affiliated with the University of Exeter Students’ Guild with a total membership of over 200 undergraduate students. Student volunteers for NPW were recruited two months in advance through marketing events during the first two weeks of the academic year. A total of 125 student volunteers were recruited and were given materials to read in preparation for their activities. The summary of student volunteers’ backgrounds is illustrated in Table 1. Students were primarily from healthcare-related courses such as Medicine, Medical Sciences and Medical Imaging.

**Table 1:** Undergraduate courses of student volunteers from the University of Exeter for Exeter National Pathology Week 2016

Undergraduate course	Number of students
Medicine (BMBS)	75
Medical Sciences (BSc)	35
Medical Imaging (BSc)	15
Total:	125

Pathologists and allied healthcare professionals from the RD&E were recruited to support the engagement activities. A total of 15 healthcare professionals involved in pathology participated throughout the week. Their backgrounds are summarised in Table 2. Their roles were to actively engage with the students to provide an insight into how pathology is involved in modern healthcare and teach basic principles in pathology.

**Table 2:** Background of pathology healthcare professionals involved in Exeter National Pathology Week 2016

Background	Number of pathology healthcare professionals
Consultant pathologist (FRCPath)	2
Specialty training pathologist	5
Academic pathologist	1
Biomedical scientist	2
Clinical scientist	3
Pathology laboratory assistant	2
Total:	15

### Funding

The Exeter Leadership & Management Society successfully obtained a £475 public engagement innovation grant scheme from the RCPath. This grant was used to support the engagement activities throughout NPW. NPW-branded merchandise including posters, pens, card holders, mini microbes and wristbands were provided by the RCPath.

### Locations and materials

Three locations were secured for the engagement activities running from 9 am to 4 pm between November 7–11, 2016. An interactive stand targeting patients was organised at the Oasis Restaurant in the Royal Devon & Exeter Hospital (RD&E). An academic stand was held at the students’ restaurant within St. Luke’s Campus at the University of Exeter targeting undergraduate students. A one-day information stand followed by an educational seminar was conducted at the Royal Cornwall Hospital (RCH) in Truro targeting medical students.

Microscopes were kindly provided by pathologists from the RD&E and Exeter Medical School. Student volunteers were trained to use microscopes to interpret histology slides by the healthcare professionals in pathology.

The student society secured interactive pathology-related workshops and presentations at nine different schools/colleges in Devon to students aged between 5 and 18. The range of topics and activities were designed to be fun and informative. These sessions were attended by student volunteers who planned and led the sessions using templates provided by RCPath online resources.

### Engagement activities

A comprehensive curriculum was created using primarily RCPath online resources for each of the locations. The theme for NPW 2016 was “*Prevention, Diagnosis and Treatment*”; the content of each stand covered each of these aspects throughout the week. The composition of student volunteers was mixed to establish a mock MDT to mimic current practice. Students were encouraged to complete further reading of the content for each day relevant to their respective course.

### Feedback

Formal feedback was obtained from both student volunteers and pathologists to provide an understanding of the impact of the public engagement model. Feedback was collected using both paper-based and online surveys to target a wide audience. Students completed a pre- and post-NPW feedback and the pathologists the post NPW feedback. Results were illustrated using bar charts and pie charts and Student’s *t* test was used to determine any significant differences between pre- and post-NPW results.

## Results

### Pre-NPW survey results

**Fig. 1:**
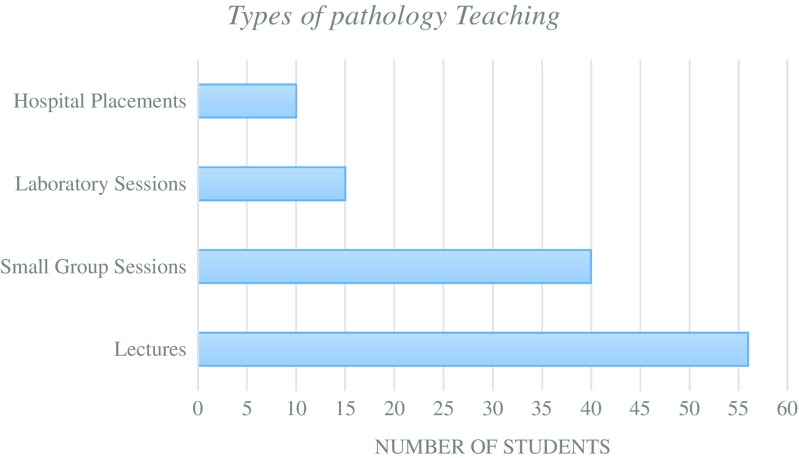
Types of pathology teaching student volunteers have experienced prior to NPW

**Fig. 2:**
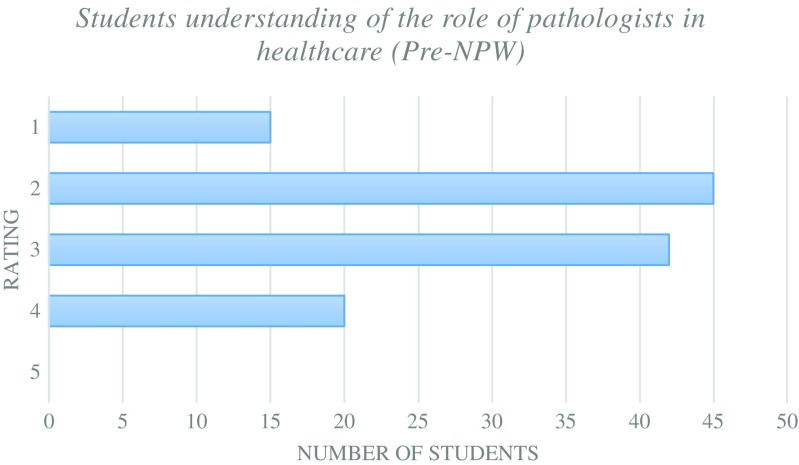
Student volunteers’ understanding of the role of pathologists within healthcare prior to NPW. Rating defined as 1 = no understanding to 5 = full understanding

**Fig. 3:**
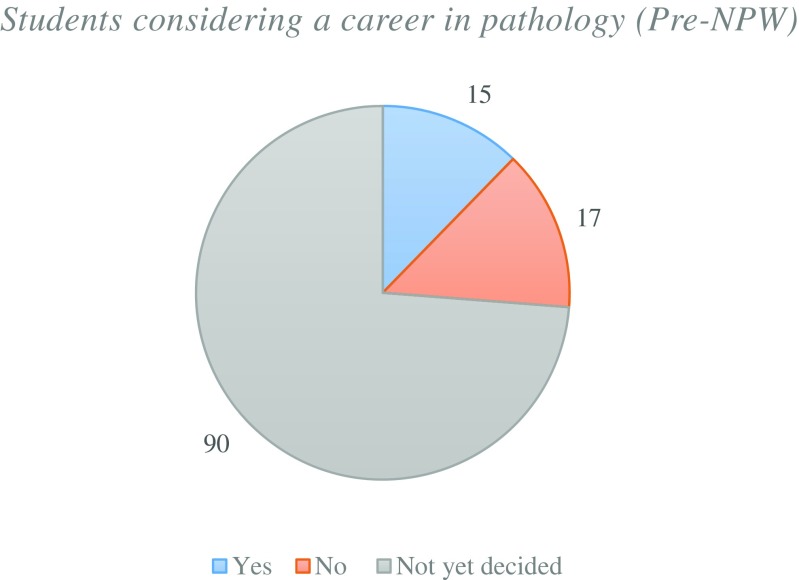
Proportion of student volunteers that are considering a career in pathology (pre-NPW)

The pre-NPW survey was designed to obtain a baseline for the student volunteers who intended to participate within NPW. The majority of students had received previous pathology teaching from lectures and small group sessions with a small number who have had laboratory experiences (Fig. 1). The students’ understanding of the roles of pathologists within healthcare was varied with the majority of students showing a low level of understanding (Fig. 2). None of the students showed a full understanding of the role of pathologists within the fields that they were currently studying. Due to this lack of understanding, the vast majority of students were still undecided on whether they considered pathology as a future career (Fig. 3). In summary, it can be assumed that the majority of student volunteers for NPW were in the early stages of their undergraduate education and were yet to complete their entire undergraduate pathology curriculum.

### Public engagement activities

**Fig. 4:**
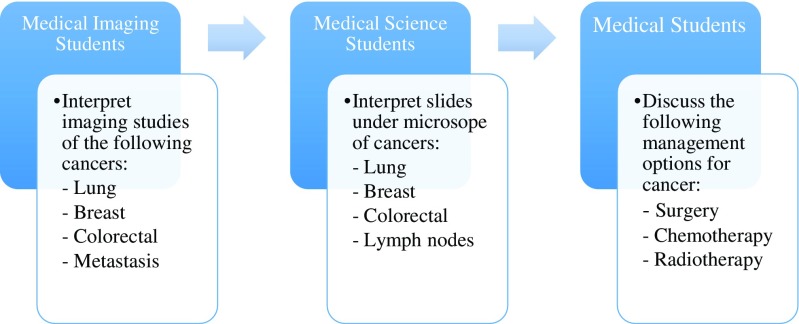
Example of how undergraduate students formed a mock multi-disciplinary team to provide an insight into how cancer is diagnosed

The Oasis Restaurant in the RDE Hospital was the primary location where students and pathologists held events throughout NPW. The events and activities organised were to engage with the public through NPW to deliver a structured curriculum that was aligned with the theme of “*Prevention*, *Diagnosis*, *Treatment*”. Over 500 members of the public interacted with the engagement stands during the week. The audience were able to obtain a number of take home messages that were changed daily to benefit returning members of public. Students from Medicine, Medical Sciences and Medical Imaging courses formed a mock MDT to provide an example of how each discipline contributes to diagnosis of cancer; this is illustrated in Fig. 4.

**Table 3:** Exeter National Pathology Week 2016 “Prevention” curriculum summarising topics and related activities

“Prevention” topics	Activities
Cervical cancer screening programme	- Information posters - CCSP myth busters - Leaflets to take away
Microscope	- Cervical cancer slides - Normal tissue slides - Quiz to diagnose cervical cancer - Quiz to identify different types of cells
Bowel cancer screening programme	- Information posters (RCPath) - BCSP myth busters - Leaflets to take away
Microscope	- Bowel cancer slides - Normal tissue slides - Quiz to diagnose bowel cancer - Quiz to identify different types of cells

#### Prevention

The first 2 days of NPW focused on how pathologists are involved in the prevention of a range of conditions summarised in Table 3. The primary learning points were focused around the cervical and bowel cancer screening programmes. Students were given preparatory materials that were summarised from the educational posters published by the RCPath. They were tasked to introduce the public to the concept of screening programmes and then summarise the inclusion criteria. Pathology slides from cervical and bowel cancer were used to allow students and the public to visualise the abnormalities. The attending pathologists were able to provide a 15–20-min teaching session to students to enable them to adequately use the appropriate terminology and identify the abnormal cells on the slides. The students were then able to consolidate their learning by teaching members of the public Table 3.

#### Diagnosis

Common pathology topics that are relevant in the wider society including inflammatory bowel disease (IBD), blood cell abnormalities and antibiotic resistance were included (Table 4). The rationale was to include topics that students would already have prior knowledge and therefore would be able to relate how pathology is involved. The majority of students had never seen pathology slides of IBD and they were fascinated to visualise inflammatory changes under the microscope. Members of the public also found these topics very interesting as they discussed the current trends in diets and how it may affect the gastrointestinal system. The topic of antibiotic resistance enabled the students to address the dilemma of antibiotic prescriptions by viewing examples of Gram staining and images of resistant bacteria on agar plates to further understand the issues.

**Table 4:** Exeter National Pathology Week 2016 “Diagnosis” curriculum topics and related activities

“Diagnosis” topics	Activities
What can pathologists diagnose?	- Coeliac disease poster (RCPath) - Pathology building blocks of life (RCPath) - Pathology myths (RCPath) - Antenatal microbiology (RCPath) - Clinical genetics of diagnosis - Immunology - Clinical biochemistry
Microscope	- Coeliac disease slides - Inflammatory cells slides - Biopsy samples (various cells) - Quiz to diagnose coeliac disease - Quiz to identify different types of cells - Bacteria under the microscope - Gram staining - Antibiotic resistance/sensitivity

#### Treatment

The final aspect of the curriculum focused on treatment following the results of a range of pathology tests and cancer screening (Table 5). Student volunteers were tasked with interpreting results of common tests including blood, faecal and urine. Mini quizzes including sample tests of various conditions and infections were available for the public to engage with. The pathologists taught students the basic concepts when assessing margins of grossed pathology samples to ensure the subsequent treatment for patients can be guided. This also enabled students from allied healthcare courses such as Medical Imaging to further understand how imaging studies must be combined with histopathology reporting to provide adequate prognostic information for patients.

**Table 5:** Exeter National Pathology Week 2016 “Treatment” curriculum topics and related activities

“Treatment” topics	Roles and activities
When do pathologists see patients?	- Infection control - Diabetes clinic - Blood transfusion - Haematology - FNA clinics - Oncology - Medical Imaging
Microscope	- Haematology samples - Vasculature samples (diabetes) - Tumour samples - Laptop screen – Medical Imaging of cancers
What is pathology?	- A-Z pathology information - What do pathologists do? - Kid’s quiz to identify organs
How does pathology affect you?	- Point of care testing poster - Common tests • Blood test • Urine test • Faecal test • Imaging - Histopathology

#### School visits

Nine schools and colleges hosted the student volunteers throughout the week to provide interactive pathology sessions. The curriculum for these sessions was based on resources provided by the RCPath summarised in Table 6. Student volunteers led a range of sessions including arts and crafts, ethical discussions and pathology quizzes. Outstanding contributions from school students were rewarded with microbe toy prizes.

**Table 6:** Exeter National Pathology Week 2016 schools and colleges presentation details

Age range	Activity name	Activity details	Duration
6–10 (primary school)	“What’s that lurking on your hands?”	Handwashing activity, using hand “bug” gels and UV lamps. Explaining the importance of handwashing through a fun workshop.	30 min
6–10 (primary school)	“Make your own bug”	Exploring the different properties of viruses and bacteria, while allowing children to design their own bug using arts and crafts materials.	30 min
14–15 (GCSE level)	“Making babies, designer babies”	Exploring the social, ethical and moral implications of assisted conception and saviour siblings. Should everyone who cannot have children be allowed to, through IVF? Should everyone be allowed to have multiple babies by IVF on NHS?	1 hr
11–16 (secondary school)	“Resistance is futile”	Microbiology and Virology: Exploring the use of antibiotics, the reasons behind antibiotic resistance and differences between viruses and bacteria.	1 hr
16–18 (A-level)	“My heart belongs to you: organ donation”	An ethical debate about organ donation. How old do you have to be to make up your own mind about something that may have a negative impact on your health? Should organ transplant go to patients who will be easier to manage or should the transplant go to the person with greater clinical need, or the one who contributes most to society?	1 hr

#### Student engagement

The academic stand at St. Luke’s Campus at Exeter Medical School followed the same NPW curriculum as the hospital stand but emphasis was placed primarily on education and careers in pathology. Booklets and information leaflets were provided by the RCPath to highlight career opportunities. Attending pathologists were tasked with providing a summary of their career progression and personal interests.

The RCH in Truro held an educational histopathology seminar led by trainee pathologists to undergraduate Medicine students. The 2-h seminar concluded with example pathology themed single best answer questions from previous examinations. Students found this concept focused and valuable as it was assessment orientated.

### Post-event survey results

#### Student volunteers

**Fig. 5:**
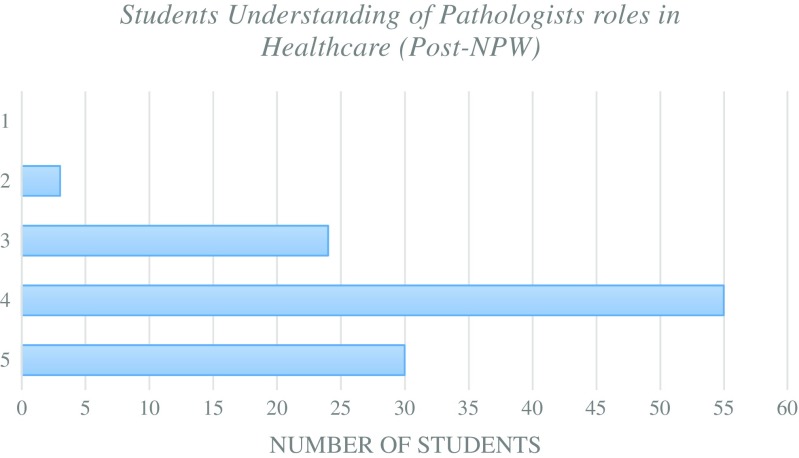
Student volunteers understanding of the role of pathologists within healthcare post NPW. Rating defined as 1 = no understanding to 5 = full understanding

One of the aims of this project was to enable students to interact with pathologists to allow them to increase their awareness of the types of careers within the field. Students showed a significant (*p = < 0.05)* increase in their understanding of the roles of pathologists within healthcare post NPW compared to baseline (Fig. 5).

**Fig. 6:**
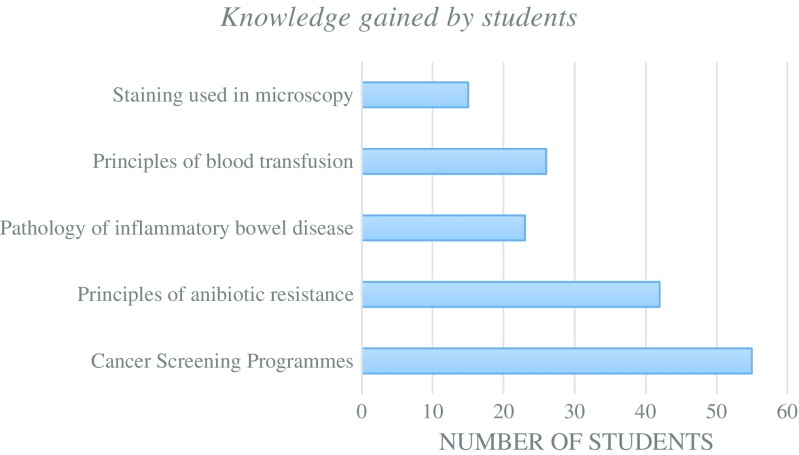
Summary of the main topics covered and knowledge gained by students during NPW

**Fig. 7:**
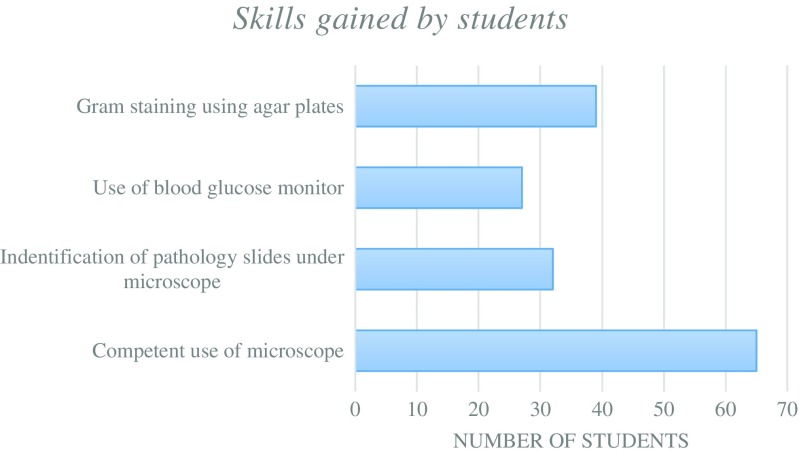
Summary of the main skills gained by students during NPW

The knowledge gained by students during NPW has been summarised in Fig. 6. The principles of antibiotic resistance and cancer screening programmes were the most common learning points as they were covered on multiple days during NPW. Similarly, the skills gained by students have been summarised in Fig. 7 showing that competent use of microscopy and gram staining were the most popular activities.

**Fig. 8:**
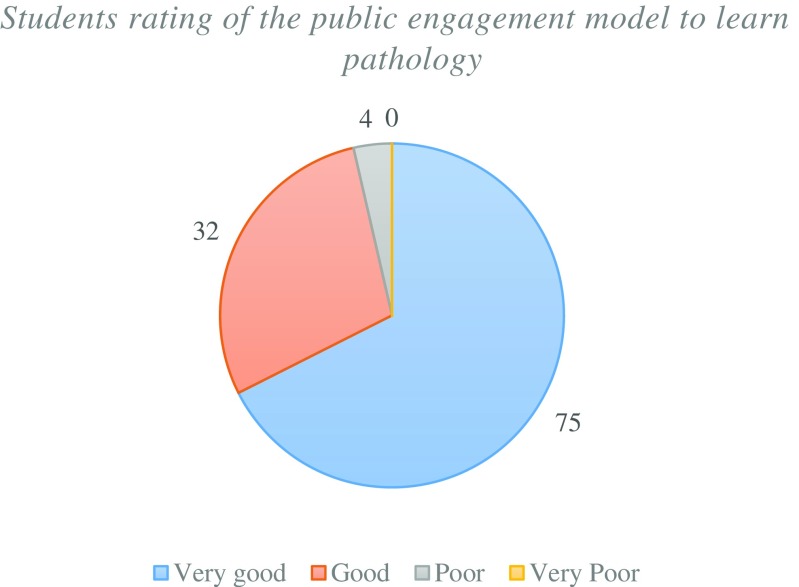
Student volunteers rating on the public engagement model within the context of pathology education

**Fig. 9:**
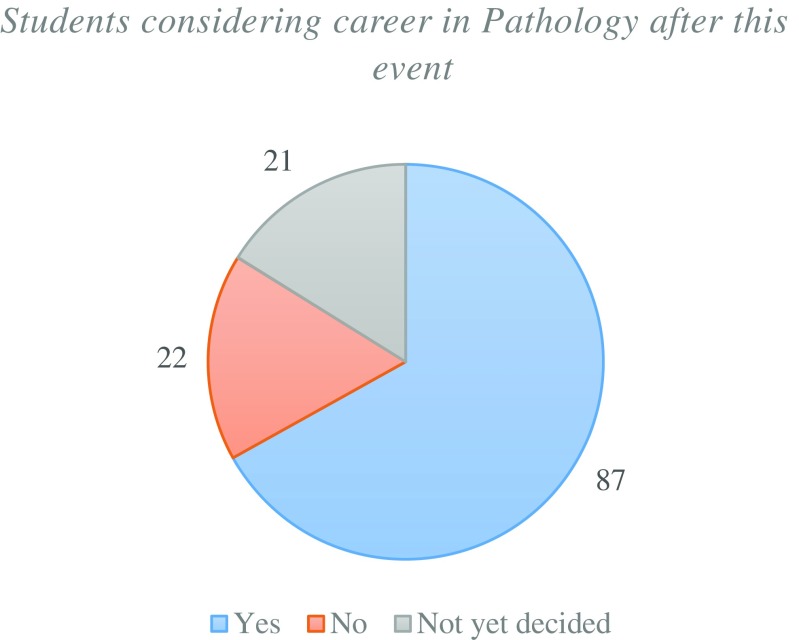
Proportion of student volunteers that are considering a career in pathology (Post-NPW)

The majority of students felt that the public engagement model was a “very good” method of delivering pathology education (Fig. 8) and this was supported by a large proportion of students now considering a career in pathology post NPW (Fig. 9). It can be therefore suggested that this model has the ability to both educate and inspire students to consider a future career in pathology.

#### Pathologists

**Fig. 10:**
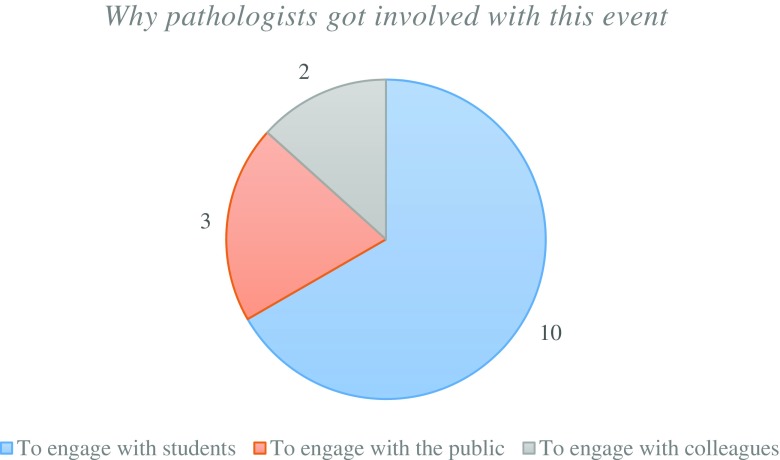
Pathologists rationale on participating in NPW

To evaluate whether the public engagement model can be developed into a formal teaching aid, it is important to consider the feedback from the pathologists. The majority of pathologists got involved with NPW with the rationale to engage with the students (Fig. 10). This can be seen as a desire to teach and educate students in their respective fields. Pathologists volunteered between 1 to 4 h of their time during NPW, with 2 h being the most common commitment. This was spread across the week and most of the pathologists used their lunch break to attend the stands.

**Fig. 11:**
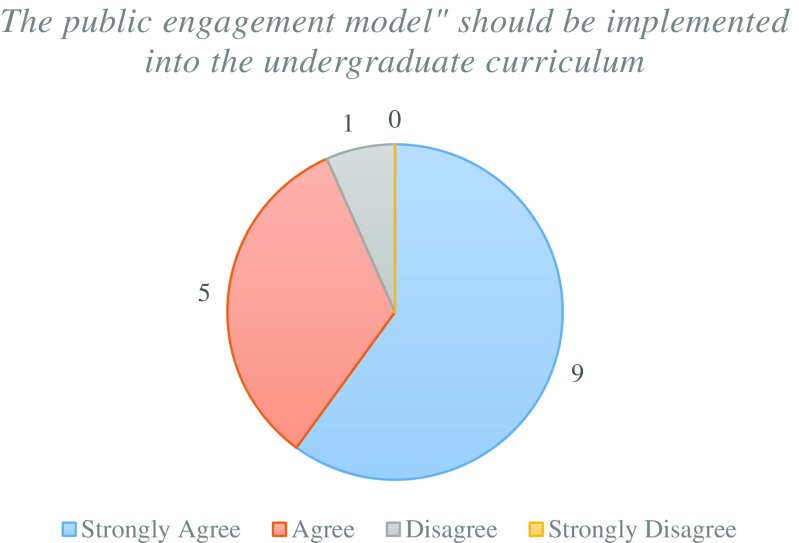
Pathologists opinion on whether the public engagement model should be implemented into the undergraduate curriculum

**Fig. 12:**
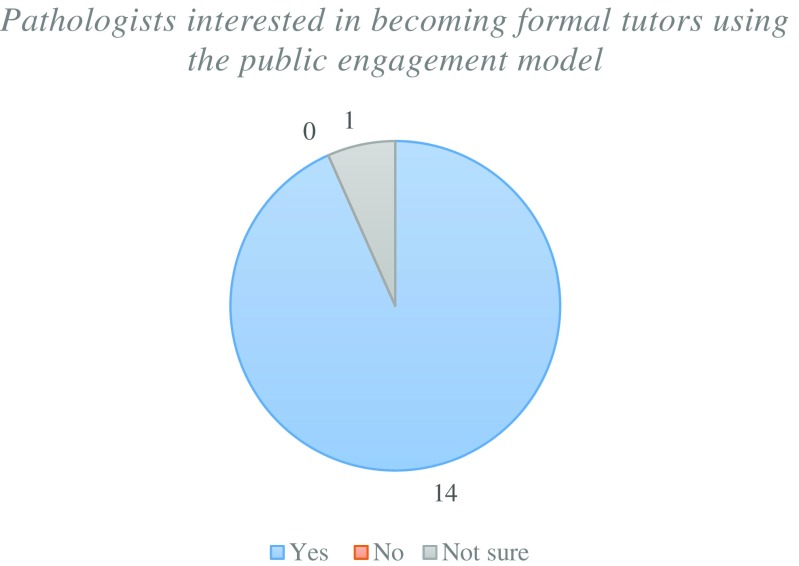
Pathologists opinion on whether they would consider working as tutors to support the public engagement model

There was an overwhelmingly positive feedback for the public engagement model with the majority of pathologists who “strongly agree” for the implementation into the undergraduate curriculum (Fig. 11). This is supported by all but one pathologist showing interest to become formal tutors for future students to support this model (Fig. 12).

## Discussion

### Innovative pathology education model

We propose public engagement as an innovative educational model to aid delivery of pathology teaching in modern medical schools. Through these events, we have successfully interacted with 125 undergraduate students who attended on a voluntary basis to learn, participate and engage in pathology-related activities. This model has a number of similarities to previously published educational theories including the adult learning theory and self-directed learning (SDL) [14]. The adult learning theory has originated from “*Andragogy*”; this term was coined by Malcom Knowles and has been established based on five assumptions of adult learning [15].

The principles that apply to the public engagement model are that students’ value learning that integrates with everyday life and the preference of problem-based approach over subject based. The NPW curriculum was introduced to the student volunteers 2 weeks prior to emphasise key learning points and the purpose of the event was emphasised. Establishing a firm rationale for their learning was instrumental for compliance, as students were required to educate the public in an appropriate and competent manner.

The preparatory materials highlighted how pathology is used in everyday clinical practice and the importance of cancer screening programmes to public health. Secondly, the daily curriculum focused on three main “problems” of “*prevention*, *diagnosis and treatment*”. This provided a logical sequence for students to apply basic knowledge of pathology to address relevant issues that concern the wider public.

The effective implementation of SDL laid the platform for this model to be applicable to students from multiple health-related courses. Students were encouraged to read and learn topics that interested them within their field to share amongst their peers and the public at the NPW stands. The effectiveness of SDL has been shown in current literature stating improvement in knowledge acquisition and retention compared to traditional teaching methods such as lectures [16]. Furthermore, the combination of SDL with peer to peer teaching via the mock MDT aspect of this model further enhances student learning experience by promoting diversity and creativity in learning [17].

Finally, the inclusion of the public and pathologists provided a platform for students to practice and implement their pathology knowledge. The “*learning by teaching*” method has been long cited in literature as an effective method of clinical education and has been utilised formally and informally by medical students worldwide [18]. Students have demonstrated knowledge retention by the repetitive explanations of the pathology topics in the curriculum to multiple members of public. The interaction with pathologists provided them an opportunity to ask questions to further their understanding of the topic and develop their own interests.

### Implementation into undergraduate medical curriculum

The implementation of the pubic engagement model into the modern undergraduate medical curriculum is not a straightforward endeavour due to the large number of existing academic and clinical commitments for students. There are a number of methods to employ aspects of the model in addition to the current pathology curriculum. These will ultimately need to undergo pilot testing and quality assurance to ensure that resources and staff can provide a consistent delivery of learning outcomes.

The public engagement model requires prior planning and organisation which requires student participation to successfully carry out events. This time commitment will be difficult to overcome for many medical students that have a rigid timetable. Student-selected components (SSC) are focused educational activities wherein medical students have 2–3 weeks to engage in tutorials and clinical placements to study a chosen speciality in medicine [19]. The academic outcome of these sessions is to produce an extended length essay focusing on the learning points they have chosen. This time period could be used to employ the public engagement model by providing students with a list of pathology topics they can focus and prepare activities. The final week of the SSC could be used to deliver the public engagement events at their local institution or hospital. The requirement of the extended essay will further strengthen this public engagement model by allowing the student to conduct focused reading of literature of their chosen pathology topics. These essays can be examined by academics and pathologists to ensure relevance and quality of the researched topic. Feedback from Fig. 12 has supported this SSC concept as the pathologists who were involved in NPW were highly motivated to become formal tutors. Junior doctors who are completing their speciality training in pathology can benefit from this model as it provides them with an opportunity to develop their teaching portfolios by supervising students.

This potential implementation of the public engagement model provides students with an interactive and rewarding experience to complement the existing pathology curriculum. The limitations of resources and ideas for events can be alleviated by referring to the RCPath website where a number of resources are freely available. NPW currently takes place in November annually; the inclusion of public engagement pathology SSC at the same time can provide further incentives for students to get involved. This can also have a profound effect on a wider scale if multiple medical schools adopt this pathology SSC concept, a cumulatively large public audience would also benefit from this scheme.

Alternative methods for implementation include reducing the scale of the events to enable students to get an introductory experience to the public engagement model. These can be delivered through small group learning sessions, lectures and seminars which students already receive. Motivated students can independently carry out public engagement events during free time following the introductory sessions. The potential issue is that many medical students are incentive and goal orientated regarding involvement in extra-curricular activities such as research and potentially the public engagement model. [20] Ultimately, there needs to be an added benefit for students to complete the public engagement activities with the required hard work and dedication. The model we have described included the involvement of a student-led society that provided participation certificates and free snacks for student volunteers to get involved. The incentive of the participation certificate signed by the president of the RCPath was enough to motivate a relatively large number of students in our project. The majority of UK medical schools have student-led societies aimed at medical students for academic and social activities. The formation of pathology-specific societies can provide a platform for future public engagement events. The organising committee of the society can liaise with the RCPath and obtain funding via the “Public Engagement Innovation Grant Scheme” and replicate the activities of previous NPW events.

Further opportunities could involve students including the public engagement model during their medical electives in their final year of study. Many students chose to go abroad for these activities and organising pathology related events in a developing country will have great impact on the public health of the region. These activities can also be conducted using the RCPath-branded resources which can be utilised to run events for “*International Pathology Day*”. The long-term sustainability of the public engagement model is dependent on the continuous innovation and refinement to ensure it can benefit the students, pathologists and the public.

It must be noted that the public engagement model has been designed to aid the teaching of pathology. The model is currently in its infancy and it cannot replace or dilute the existing undergraduate pathology curriculum. It is also difficult to cover all sub-specialities of pathology and the entire undergraduate curriculum with this model as it is designed to be applicable to the wider public from children to the elderly. Therefore, it should currently be used to emphasise the current topics in pathology that are widely discussed in the media to allow both students and public to increase their awareness.

The short-term benefits of this model will allow undergraduate students to learn key concepts in pathology whilst developing skills such as leadership and project management. In the longer term, we hope that this experience ignites stronger interests in pathology for students who may eventually pursue careers in the speciality.

## Conclusion

We have proposed an innovative public engagement model to further develop the undergraduate pathology curriculum and rejuvenate interest in the speciality. The activities and events organised during Exeter NPW 2016 were unique in that they were entirely student led.

The model reflects current successful theories of medical education and has the potential to integrate medical students with allied healthcare courses to facilitate peer to peer learning. The implementation of the model into the medical curriculum is possible in the form of SSC and informative seminars and student-led societies to provide the platform for greater numbers of student participation.

Further refinement and development of the model could lead to integration into a wider range of undergraduate courses that involve pathology, such as Biological Sciences, Microbiology and Biomedical Sciences. Such innovative educational models are required for the long-term sustainability of pathology as a discipline and thus to ensure the health of the community.
